# Acute Encephalitis in an Adult with Diffuse Large B-Cell Lymphoma with Secondary Involvement of the Central Nervous System: Infectious or Non-Infectious Etiology?

**DOI:** 10.3390/jcm6120117

**Published:** 2017-12-07

**Authors:** Surinder S. Moonga, Kenneth Liang, Burke A. Cunha

**Affiliations:** 1 Stony Brook School of Medicine, State University of New York, Stony Brook, NY 11790, USA; surinder.moonga@stonybrookmedicine.edu; 2 Independent Scholar, 54 Catherine Street, New York, NY 10038, USA; liang.kenneth@outlook.com; 3 Infectious Disease Division, NYU-Winthrop University Hospital, Mineola, NY 11501, USA

**Keywords:** neuropsychiatric presentation of encephalitis, paraneoplastic encephalitis, autoimmune encephalitis, infectious encephalitis, diffuse large B-cell lymphoma

## Abstract

Both infectious and non-infectious etiologies of acute encephalitis have been described, as well as their specific presentations, diagnostic tests, and therapies. Classic findings of acute encephalitis include altered mental status, fever, and new lesions on neuroimaging or electroencephalogram (EEG). We report an interesting case of a 61-year-old male with a history of diffuse large B-cell lymphoma with secondary involvement of the central nervous system (SCNS-DLBCL). He presented with acute encephalitis: altered mental status, fever, leukocytosis, neuropsychiatric symptoms, multiple unchanged brain lesions on computed tomography scan of the head, and EEG showed mild to moderate diffuse slowing with low-moderate polymorphic delta and theta activity. With such a wide range of symptoms, the differential diagnosis included paraneoplastic and autoimmune encephalitis. Infectious and autoimmune/paraneoplastic encephalitis in patients with SCNS-DLBCL are not well documented in the literature, hence diagnosis and therapy becomes challenging. This case report describes the patient’s unique presentation of acute encephalitis.

## 1. Introduction

The substantial morbidity and mortality that is associated with encephalitis is well documented in the literature, although the specific etiology is not often well characterized. Among the identified etiologies, the most commonly described is of infectious nature, either through direct effects on the brain parenchyma or by post-infectious processes, including acute disseminated encephalomyelitis (ADEM) and acute hemorrhagic leukoencephalitis [1,2]. Viruses are the most common cause of encephalitis, with herpes simplex virus (HSV) being the most common [3]. Non-infectious etiologies are typically immune-mediated, divided between autoimmune pathogenic autoantibodies against surface neuronal antigens and paraneoplastic onconeural antibodies (most common Hu and Ma2) against intracellular neuronal antigens [4,5].

Boucher et al. revealed an estimated annual incidence of infectious encephalitis to be 1.5–7/100,000 [3]. A United Kingdom-based prospective surveillance study found the annual incidence of non-infectious autoimmune encephalitis to be 0.85/1,000,000 in children [6].

Per the International Encephalitis Consortium held in March 2012, the major criterion for the diagnosis of encephalitis includes altered mental status lasting ≥24 h without an otherwise identifiable cause [7]. Minor criteria for diagnosis include: temperature ≥ 100.4 °F (38 °C), new-onset seizure, new focal neurological abnormalities, cerebral spinal fluid (CSF) white blood cell (WBC) ≥ 5/mm^3^, acute lesion on neuroimaging, or abnormality on electroencephalogram (EEG) indicative of encephalitis [7]. In an EEG study of 52 patients with diagnosed encephalitis, 96.2% demonstrated generalized or focal slowing, 50% showed abnormal delta activity, and 46% of patients showed abnormal theta activity [8]. Overall, EEG rhythm disorder recordings were mild, moderate, and severe for 40.4%, 44.2%, and 13.5% of patients, respectively [8].

Not surprisingly, the neurologic manifestations of encephalitis are most commonly described in the literature; however, on rare occasions, psychiatric pathology can be the only clinical symptom marking the initial presentation of encephalitis, specifically in non-infectious, autoimmune encephalitis [9]. Psychiatric manifestations in non-infectious, autoimmune encephalitis have a well-documented association with autoantibodies against anti-*N*-methyl-d-aspartate receptor (NMDAR), α-amino-3-hydroxy-5-methyl-4-isoxazolepropionic acid receptor (AMPAR), and γ-aminobutyric acid receptor (GABAR) [10]. Symptoms of psychosis may include paranoid delusions and visual/auditory hallucinations; additionally, patients may also present with insomnia, confusion, and short-term memory loss, similar to delirium [10,11]. The CSF in these non-infectious cases may show lymphocytic pleocytosis and increased protein; further analysis of the CSF can show antibodies to the aforementioned autoantibodies [12]. An increased T2 signal of the medial temporal lobe on magnetic resonance imaging (MRI) or the complete absence of cerebral pathology can be seen on neuroimaging of patients with these autoantibodies [10].

We report an interesting case of acute encephalitis in a 61-year-old male with history of diffuse large B-cell lymphoma (DLBCL) with secondary involvement of the central nervous system (SCNS-DLBCL). Very few cases of encephalitis that are associated with DLBCL have been reported in the literature, and considerations of infectious versus non-infectious etiologies in this particular clinical scenario are not well addressed. Furthermore, previously documented cases of acute encephalitis in patients with DLBCL have not specified secondary lymphomatous central nervous system (CNS) involvement. Was the patient’s presentation of altered mental status, fever, leukocytosis and neuropsychiatric symptoms secondary to infectious causes, or were non-infectious, paraneoplastic, or autoimmune processes responsible?

## 2. Case Report

A 61-year-old male presented to the Emergency Department (ED) with altered mental status and fever after a follow-up lumbar puncture (LP) one day prior. 18 h after the procedure, the patient’s wife noted that he was “shaking in his wheelchair, mumbling to himself, unresponsive and staring into space”. She recorded a home temperature of 99 °F. The patient has a past medical history of SCNS-DLBCL (diagnosed two years ago with right axillary lymph node biopsy and LP with CNS cytology, unknown location of primary tumor), associated with warm autoimmune hemolytic anemia, that has received multiple trials of chemotherapy and improved, but has not entered remission.

Of note, this patient was previously hospitalized two months ago for similar symptoms, including aphasia, lethargy, and right hemiparesis; he was found to have worsening SCNS-DLBCL involvement on computed tomography (CT) scan of the head. Imaging revealed significant left temporal lobe, left parietal lobe, and right occipital lobe lesions, with leptomeningeal involvement in the medial right parietal region. The patient clinically stabilized following whole brain radiation. A follow-up MRI (one month prior to this hospitalization) showed a marked improvement of CNS infiltration and disease. The patient saw his neuro-oncologist five days prior to hospitalization, who stated that the patient’s neurologic symptoms, including his memory, had greatly improved. The patient’s other medical problems include a urinary tract infection (UTI) that was diagnosed two weeks prior to admission (treated with cefadroxil) and likely steroid-induced hyperglycemia. The patient has never been diagnosed with any psychiatric disorder, has never taken any psychiatric medication, and has never been admitted to a psychiatric hospital. However, the patient’s wife mentioned that he had been more isolative in the past few weeks, less talkative, and easily angered. She denies noting paranoia or any complaints of auditory/visual hallucinations from the patient.

On admission, the patient was febrile (102.1 °F, 38.94 °C) with sinus tachycardia (125 beats/min). His physical exam was notable for altered mental status (oriented to self only), generalized weakness, and unspecified aphasia. Pertinent negatives included: no neck stiffness, photophobia, vomiting, respiratory distress, recent trauma, or headache. Medications on admission included: dexamethasone 1 mg daily, long-acting insulin 11 units at bedtime, levetiracetam 500 mg twice/day, metformin 1000 mg daily, pantoprazole 40 mg daily, and ibrutinib 560 mg daily. The patient had no known drug allergies.

Admission laboratory results included: leukocytosis, WBC count = 16.6 K/μL (neutrophils 77%, stabs 3%, lymphocytes 17%, monocytes 9%), venous blood gas lactate of 3.3 mmol/L (*n* = 0.5–2.2 mmol/L), and normal chemistry with the exception of elevated blood urine nitrogen (BUN 25 mg/dL, creatinine 0.8 mg/dL). Other laboratory results include: normal aspartate aminotransferase (AST) of 13 IU/L (*n* = 4–36), normal alanine aminotransferase (ALT) of 13 IU/L (*n* = 13–39), elevated C-reactive protein (CRP) of 76.98 mg/L (*n* < 3), normal sedimentation rate (9 millimeters/h (MM/HR), *n* < 20 MM/1HR), elevated ferritin (1593 ng/mL, *n* = 14–235 ng/mL), normal thyroid stimulating hormone (TSH) (4.136 μIU/mL, *n* = 0.36–5.80 μIU/mL), and thyroxine (T4) (1.27 ng/dL, *n* = 0.89–1.76 ng/dL). Patient’s urinalysis was positive for WBC > 182 (*n* < 3/high power field (HPF)), 2+ urine protein, 1+ occult blood, 3+ leukocyte esterase, and positive nitrite. Patient’s CSF analysis from LP two days prior was within normal limits.

The patient’s concerning symptoms on evaluation necessitated repeat LP, which was performed one day after admission. CSF analysis was significant only for elevated protein (51 mg/dL, *n* = 8–32 mg/dL) and appearance was non-xanthochromic and clear. The remaining CSF analysis showed 0/μL WBCs, 13/µL red blood cell (RBC), and 2.2 mmol/L lactic acid. CSF was negative for Cytomegalovirus polymerase chain reaction (PCR), Epstein Barr Virus PCR, HSV-1 PCR, HSV-2 PCR, Herpes 6 Virus IgG antibody and West Nile IgG/IgM. CSF cytology was non-diagnostic and flow cytometry was negative for the involvement of lymphoma. The patient was discharged before consideration arose to test his CSF for paraneoplastic or autoimmune antibodies. CSF culture with Gram stain showed no growth for three days.

**Figure 1:**
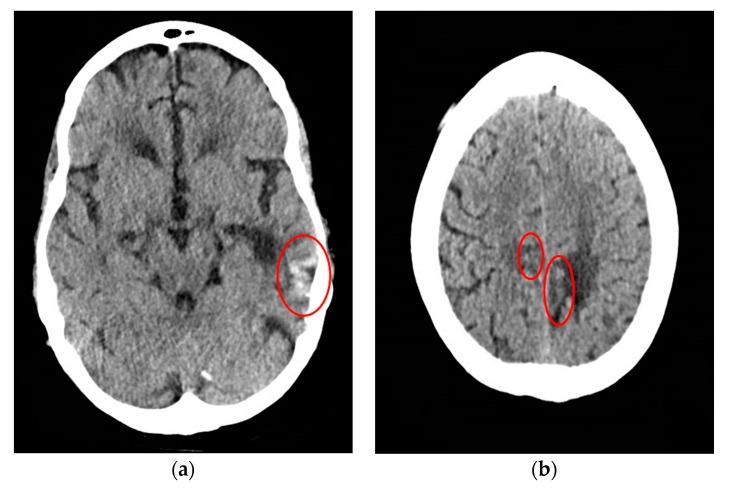
Computed tomography (CT) head of a 61-year-old male with SCNS-DLBCL presenting with acute encephalitis. (**a**) CT Head shows a small area of inferolateral left temporal density, which correlates with the blood products that were seen in patient’s previous magnetic resonance imaging (MRI) associated with the temporal lobe lesion. There is associated white matter low density, which is similar to the non-enhancing signal on previous MRI; (**b**) CT Head shows a small area of medial left parietal occipital low density corresponds to the lesion and old blood seen on the MRI. It also shows a very small area of medial right parietal occipital low density corresponds to signal seen on the MRI.

**Figure 2:**
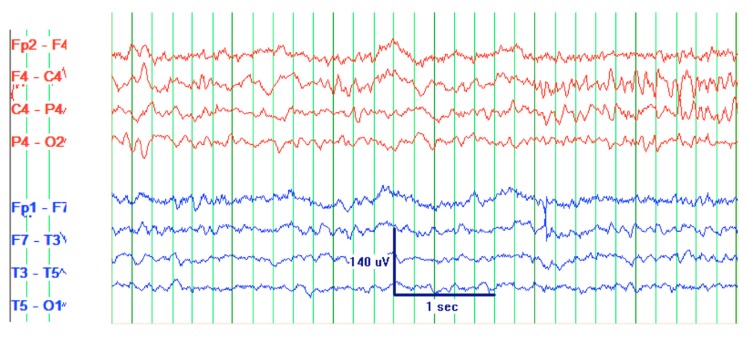
Electroencephalogram (EEG) of a 61-year-old male with SCNS-DLBCL presenting with acute encephalitis. Consistent with an abnormal EEG reading due to mild-moderate diffuse slowing. This specific frame shows excess low to moderate voltage (15 to 40 uV) polymorphic delta and theta activity. Interpretation of this EEG suggests mild to moderate diffuse cerebral dysfunction. Technical information: Electrodes were placed according to the 10–20 International Electrode System. Digital EEG was recorded using the Natus Digital EEG System and EEG was reformatted into multiple montages as needed.

CT scan of the head was reassuring, showing no significant changes from the patient’s previous MRI, performed one month prior to this hospitalization. Unchanged lesions include: small inferolateral left temporal density, associated white matter low density, small medial right and left parietal occipital low densities, and a posterior cerebral white matter hypodensity (Figure 1). Patient’s EEG result read: abnormal EEG due to mild to moderate diffuse slowing, with excess low-moderate voltage polymorphic delta and theta activity (Figure 2).

The patient’s clinical presentation of acute altered mental status, fever, and generalized weakness, in combination with temporally related recent LP, abnormal EEG, and history of SCNS-DLBCL, suggests multiple different diagnoses, including acute encephalitis. This was added to the list of differential diagnoses, which included sepsis, meningitis, and delirium secondary to UTI. Therefore, the patient was treated with 1 dose of IV vancomycin 1 g and 2 doses at 1.5 g; 1 dose of IV meropenem 1 g and 4 doses at 2 g; and, 2 doses of oral (tablet) dexamethasone 1 mg. The patient also received 1 L of IV normal saline on admission.

Treatment resulted in resolution of fever and leukocytosis (98.3 °F and 4.7 K/μL WBC upon discharge). The patient was discharged appearing clinically stable; however, his neuropsychiatric impairments revealed on admission were unresolved. The patient remained disoriented to place and time, aphasic, and emotionally distressed by word recall disturbances. Initially, the patient’s presentation was suggestive of memory impairment given his reluctance and anger toward questions that tested memory. Further examination with Montreal Cognitive Assessment (MoCA) score of 7/30 suggested profound memory loss and cognitive disturbances (Table 1) [13]. Additionally, the patient had several bizarre mannerisms in speech and behavior, sometimes speaking in paradoxes, e.g., “I am afraid, but I have no fear, is that not strange?” Mental status exam before discharge revealed: guarding throughout the interview, soft speech with regular rhythm and prosody, and blunted affect with anxious qualities. The patient appeared somewhat internally preoccupied and paranoid at times, although he specifically denied persecutory delusions and auditory/visual hallucinations. Motor behavior was significant for prominent psychomotor agitation. Overall, he demonstrated poor insight into his condition and poor judgment about the need for hospitalization, stating multiple times “there is no reason for me to be here, I am being held against my will.”

## 3. Discussion

Our patient with SCNS-DLBCL presented with symptoms of altered mental status, aphasia, fever, leukocytosis, and multiple neuropsychiatric symptoms. This clinical presentation yielded a wide differential diagnosis, including encephalitis, sepsis, meningitis, increased CNS tumor burden, and delirium. Appropriate procedural, imaging, and laboratory testing was performed. An MRI brain should have been considered to check for cerebral ischemia; however, this diagnosis was not on the differential at the time of admission. Though the patient presented after an LP, the absence of a stiff neck and negative CSF findings eliminated acute bacterial meningitis in the differential. Although the patient’s presentation of dehydration and elevated CRP support the diagnosis of sepsis secondary to a UTI, the resolution of symptoms was not achieved with rehydration and the appropriate antibiotic therapy [14]. Regarding delirium, the patient had multiple risk factors, including underlying brain injury from his cancer and an active source of infection (UTI) [15]. Additionally, the disturbances in consciousness and diffuse slowing on EEG were consistent with delirium [16]. However, the clinical picture of ‘waxing-waning’ cognition and hypoactive/agitated states that classically characterizes delirium was not present in our patient [17].

Acute encephalitis was our diagnosis, given that our patient met the criteria of the International Encephalitis Consortium: major criteria of altered mental status and minor criteria of fever (102.5 °F), new abnormal EEG findings that were consistent with encephalitis and new focal neurological deficits, presenting as aphasia and psychiatric abnormalities on mental status examination [6]. We suggest that the patient’s acute psychiatric presentation should be considered a focal neurological deficit, as it can be directly linked to the perturbations in the temporal lobe resulting in symptoms of fear, profound memory loss, and internal preoccupation [18,19,20]. Per the patient’s neuro-oncologist, these symptoms were not present five days prior to our evaluation. Furthermore, the EEG findings of diffuse background slowing and abnormal delta/theta activity are indicative of encephalitis [7,8].

Extensive CSF analysis lowered infectious encephalitis on the differential. Additional CSF testing for Zika, chikungunya and dengue viruses was not performed. Acevedo et al. recommend multiplex real-time reverse transcription PCR (rRT-PCR) testing for patients in endemic areas presenting new neurological symptoms [21]. We believe that the acute change in fear, profound memory impairments, and mild internal preoccupation are suggestive of limbic encephalitis. However, we are not confident in labeling the patient with limbic encephalitis, given that temporal lobe inflammation is confounded by the patient’s preexisting mass from SCNS-DLBCL in that region. The cost of ordering paraneoplastic/autoimmune autoantibodies tests is high, and testing all patients with SCNS-DLBCL with possible encephalitis may be impractical [22]. Therefore, we suggest a thorough examination of the neuropsychiatric symptoms of patients with SCNS-DLBCL presenting with acute encephalitis to prompt investigation of non-infectious etiologies.

## 4. Conclusions

In an adult with diffuse large B-cell lymphoma with secondary involvement of the central nervous system presenting with altered mental status, fever, and neuropsychiatric symptoms, it is imperative that clinicians consider encephalitis, both infectious and non-infectious etiologies.

## Figures and Tables

**Table 1:** Patient Montreal Cognitive Assessment (MoCA) test assessment result of 7/30 with good effort.

MoCA	
Visuospatial/executive function	0/5, patient attempted a digital clock at first, could not put numbers on circle clock
Naming	3/3
Memory	Unscored, able to repeat 5/5 words in both 1st and 2nd trials
Attention	2/2 for repeating digits forward and backward 0/1 attending to the letter A in a list (9 errors), and 0/3 serial 7’s
Language	2/2 repeating phrase 0/1 naming > 11 words that begin with the letter “F” in 1 min (named 3 words)
Abstraction	0/2
Delayed recall	0/5 with no cue 0/5 with category cue 3/5 with multiple choice cue
Orientation	0/6 (date, month, year, day, place, city)
Final score	7/30

Score = *n*; *n* ≥ 26/30 (normal), *n* ≤ 23/30 (mild cognitive impairment), *n* ≤ 17/30 (mild dementia), *n* ≤ 9/30 (moderate dementia).
